# TickNET—A Collaborative Public Health Approach to Tickborne Disease Surveillance and Research

**DOI:** 10.3201/eid2109.150301

**Published:** 2015-09

**Authors:** Paul Mead, Alison Hinckley, Sarah Hook, C. Ben Beard

**Affiliations:** Centers for Disease Control and Prevention, Fort Collins, Colorado, USA

**Keywords:** Ticks, tickborne disease, Lyme disease, anaplasmosis, ehrlichiosis, babesiosis, surveillance, prevention, bacteria, viruses, parasites, Emerging Infections Program, EIP

## Abstract

TickNET, a public health network, was created in 2007 to foster greater collaboration between state health departments, academic centers, and the Centers for Disease Control and Prevention on surveillance and prevention of tickborne diseases. Research activities are conducted through the Emerging Infections Program and include laboratory surveys, high-quality prevention trials, and pathogen discovery.

Through their bites, ticks expose humans to a remarkable array of pathologic agents, including neurotoxins, allergens, bacteria, parasites, and viruses. The clinical features of tickborne illness range from mild to life-threatening, and collectively, tickborne diseases constitute a substantial and growing public health problem in the United States. New agents of tickborne disease are described regularly, and known agents are spreading to new areas.

The most common tickborne disease in the United States is Lyme disease, caused by the spirochete *Borrelia burgdorferi*. With >37,000 cases reported to the Centers for Disease Control and Prevention (CDC) during 2013, Lyme disease ranks fifth among all nationally notifiable conditions (*1*,*2*). Less common but potentially serious tickborne infections include anaplasmosis, babesiosis, ehrlichiosis, spotted fever group rickettsioses, and Powassan virus disease (*3*). Recent reports of US patients infected with *Borrelia miyamotoi* (*4*), an *Ehrlichia muris*–like agent (*5*), a novel bunyavirus (*6*), and a putative new genospecies of *Borrelia burgdorferi* (B. Pritt, pers.com.) all serve to highlight the potential for discovery of novel tickborne pathogens. In addition, several tickborne diseases of unknown etiology have also been described, most notably STARI (southern tick–associated rash illness). Easily confused with early Lyme disease, STARI is a distinct, idiopathic entity associated with bite of the lone star tick, *Amblyomma americanum* (*7*,*8*). This tick species has also been implicated recently as a cause of IgE-mediated hypersensitivity to red meat and certain chemotherapeutic agents (*9*).

Tickborne diseases pose special challenges for clinicians and public health agencies alike. Although tickborne diseases occur throughout the United States, the distribution of any given disease can be highly focal (Figure 1), and this information must be known and considered by health care providers when assessing patients. In addition, laboratory testing is often limited to serologic assays that require paired samples drawn several weeks apart to confirm recent infection, which complicates the use of laboratory testing for both patient management and public health surveillance. With regard to prevention, tick checks, repellent use, and other personal protective measures, although generally benign and inexpensive, are not especially effective (*10*). Despite decades of education about these measures, case reports for the more common tickborne diseases continue to increase (Figure 2). Pesticide use can reduce tick abundance (*11**–**13*) but has not been proven to reduce tickborne disease in humans (*14*,*15*). Lymerix, developed to prevent Lyme disease, is the only vaccine ever licensed in the United States to prevent a tickborne disease in humans, but it was removed from the market during 2003 amidst poor sales and unsubstantiated reports of increased adverse events (*16**,**17*).

**Figure 1:**
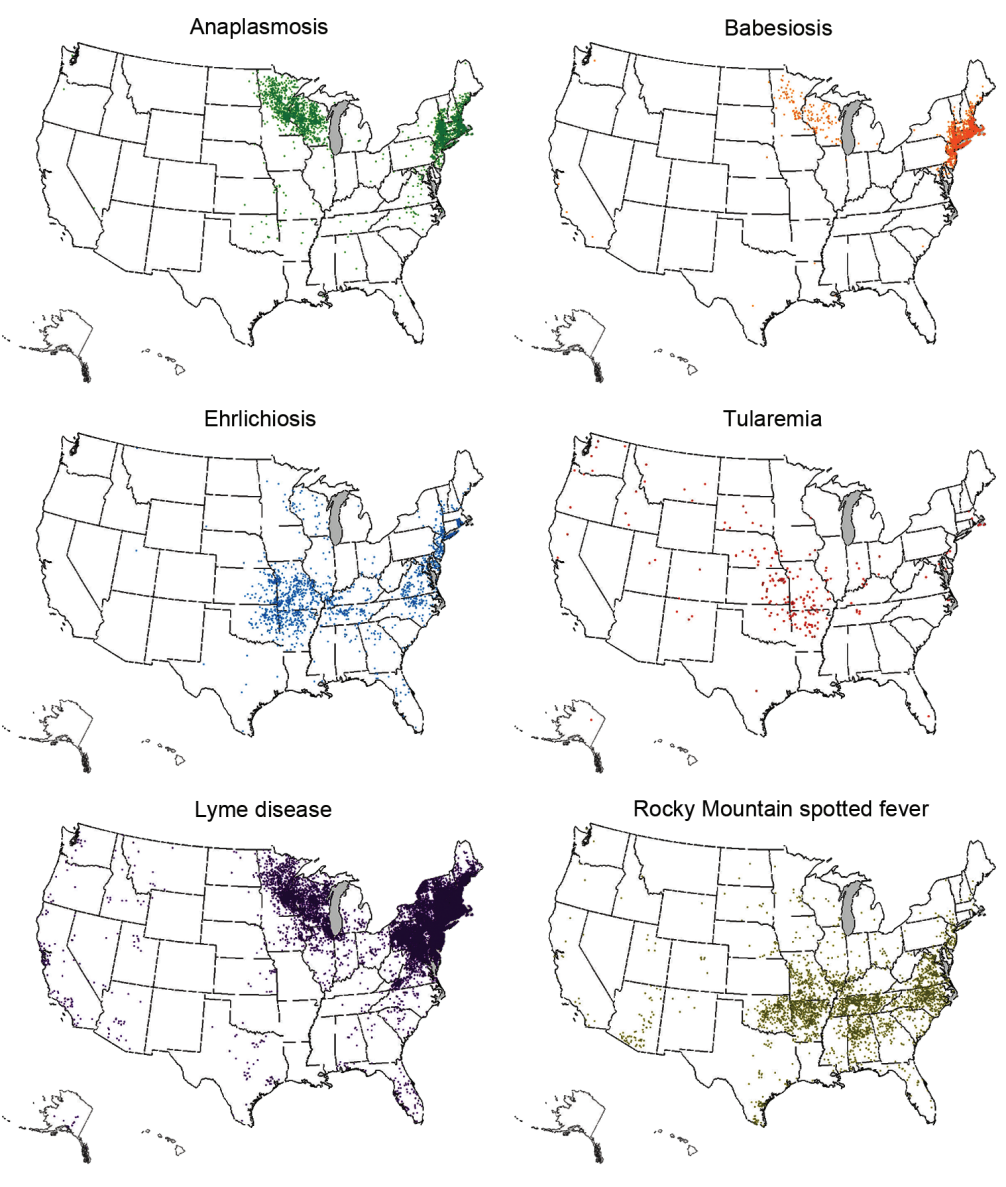
Geographic distribution of leading tickborne diseases among humans, United States, 2013. Each dot represents 1 case, based on patient residence; exposure location may be different.

**Figure 2:**
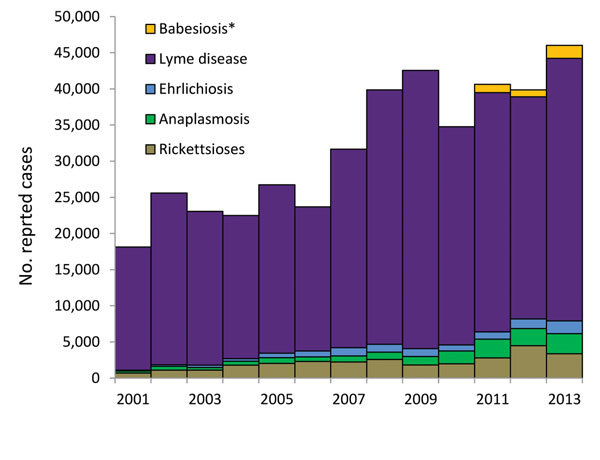
US cases of Lyme disease, ehrlichiosis, anaplasmosis, babesiosis, and spotted-fever group rickettsioses reported to the Centers for Disease Control and Prevention, 2001–2013. Counts include confirmed and probable cases, according to the case definition in effect in each year. Anaplasmosis cases were reported as human granulocytic ehrlichiosis before 2008. Ehrlichiosis refers to infections caused by *Ehrlichia chaffeensis*, *E. ewingii*, and undetermined species. *Babesiosis was first designated a nationally notifiable condition during 2011.

## The Network

To foster greater coordination on surveillance, research, education, and prevention of tickborne diseases, CDC established TickNET during 2007. TickNET is a public health network that includes partners from state health departments and academic institutions collaborating through the Emerging Infections Program (EIP), staff of state and local health departments collaborating through the Epidemiology and Laboratory Capacity (ELC) cooperative agreement, and CDC staff in the Division of Vector-Borne Diseases and the Division of Parasitic Diseases and Malaria. We will briefly describe key TickNET projects completed or currently underway.

TickNET provides funding to state and local health departments through the ELC cooperative agreement to help sustain and enhance routine surveillance for tickborne diseases. Approximately 18 state and local health departments are funded annually for Lyme disease surveillance, with priority given to states with a reported incidence of Lyme disease greater than the national average and to bordering states where the disease may be spreading. During 2014, an additional 7 state and local health departments received ELC funding to support surveillance for other tickborne diseases.

Together with ELC funding for program support, funding through EIP has allowed TickNET partners in Maryland, Minnesota, and New York to undertake special studies to quantify underreported tickborne diseases. These studies include a review of patient charts and codes from the International Classification of Diseases, Ninth Edition, and provide insights into the use of electronic medical records for public health surveillance. Other studies in Massachusetts, Minnesota, and New York are examining ways to streamline the evaluation of positive laboratory reports by using random sampling methods. Results from these and related studies will become available in 2015.

During 2008, TickNET partners at EIP sites in Connecticut, Maryland, Minnesota, and New York conducted a survey of commercial, clinical, and state laboratories to evaluate practices and volume of testing for 5 leading tickborne diseases. Collectively, 7 large commercial laboratories reported testing ≈2.4 million patient specimens for evidence of *B. burgdorferi* infection during 2008, at an estimated cost of $492 million. After correcting for test sensitivity, specificity, and stage of illness, the overall frequency of infection among patients for whom samples were tested was estimated at ≈12%. Applied to the total number of specimens, this percentage yielded an estimated 288,000 true *B. burgdorferi* infections (range 240,000–444,000) among source patients during 2008 (*18*). Results of this study will be compared with results of other ongoing CDC studies to estimate the overall frequency of Lyme disease and other tickborne infections in the United States.

Frequency is but one measure of the public health importance of a disease. To better quantify the public health burden of tickborne diseases, TickNET EIP partners in Connecticut, Maryland, Minnesota, and New York have undertaken a study to quantify current costs associated with individual cases of Lyme disease. Begun during 2014, the Cost of Lyme Disease study uses a prospective survey design to capture individual and societal costs of Lyme disease, including out-of-pocket medical costs, nonmedical costs, and productivity losses, as well as total direct medical costs to society by using billing codes from enrolled patients’ providers. This estimate will be used to guide impact assessments of current and future prevention methods.

As an adjunct to personal protective measures such as use of insect repellents, several yard-based interventions have been proposed to reduce tick abundance in the home environment. To assess the efficacy of such interventions in preventing human illness, TickNET sites have insti­tuted a series of studies to evaluate the efficacy of novel and commercially available prevention strategies. One study, a randomized, blinded, placebo-controlled, multi­state trial assessing the effectiveness of acaricide barrier sprays, will cover ≈2,700 households in 3 states, with out­comes measures including tick density on acaricide-treated properties, the number of tick–human encounters, and the number of tickborne diseases in humans. (Study results are forthcoming.) A second study, begun in Connecticut dur­ing 2012, uses a similar design to evaluate the effective­ness of bait boxes that apply fipronil to rodents that are the reservoirs of *B. burgdorferi*. Used by veterinarians to pre­vent flea and tick infestations on dogs, fipronil kills ticks on the rodents for several weeks and may potentially interrupt the local transmission cycle of *B. burgdorferi*. This study of 625 enrolled households will be completed during 2016.

Recent experience indicates that additional tickborne pathogens are waiting to be discovered. In collaboration with the Tennessee and Minnesota health departments, the Mayo Clinic, and Vanderbilt University, TickNET has recently initiated a study to identify novel agents of tickborne disease. Over the next 3 years, >30,000 clinical specimens from US patients with suspected tickborne diseases will be screened by using high-throughput molecular methods designed to detect bacteria, followed by use of genomic sequencing to characterize detected pathogens. The ultimate goal is to better describe the epidemiologic and laboratory features associated with recognized and novel tickborne pathogens and to guide the development of new diagnostic methods.

## Conclusions

Although sometimes overlooked, tickborne diseases pose an increasing threat to public health. Factors driving the emergence of tickborne diseases are poorly defined, but current prevention methods are clearly inadequate. Addressing this problem requires a multidisciplinary approach with input of entomologists, epidemiologists, educators, and infectious disease and communications specialists. Built on the pillars of the EIP and the ELC cooperative agreements, TickNET provides a collaborative network that brings together these resources at the federal and state levels to enhance surveillance, improve prevention, and identify new tickborne diseases.
